# Multiple Chemical Sensitivity Syndrome: A Principal Component Analysis of Symptoms

**DOI:** 10.3390/ijerph17186551

**Published:** 2020-09-09

**Authors:** Antonio Del Casale, Stefano Ferracuti, Alessio Mosca, Leda Marina Pomes, Federica Fiaschè, Luca Bonanni, Marina Borro, Giovanna Gentile, Paolo Martelletti, Maurizio Simmaco

**Affiliations:** 1 Department of Dynamic and Clinical Psychology, Faculty of Medicine and Psychology, Sapienza University, 00185 Rome, Italy; 2 Unit of Psychiatry, ‘Sant’Andrea’ University Hospital, 00189 Rome, Italy; 3 Department of Human Neuroscience, Faculty of Medicine and Dentistry, Sapienza University, 00185 Rome, Italy; stefano.ferracuti@uniroma1.it; 4 Unit of Risk Management, ‘Sant’Andrea’ University Hospital, 00189 Rome, Italy; 5 Department of Neuroscience, Imaging and Clinical Sciences, “G. d’Annunzio” University, 66100 Chieti, Italy; alessio.mosca909@gmail.com; 6 Department of Neuroscience, Mental Health, and Sensory Organs (NESMOS), Faculty of Medicine and Psychology, Sapienza University, 00185 Rome, Italy; ledama@hotmail.it (L.M.P.); federica.fiasche@uniroma1.it (F.F.); luca.bonanni4@gmail.com (L.B.); marina.borro@uniroma1.it (M.B.); giovanna.gentile@uniroma1.it (G.G.); maurizio.simmaco@uniroma1.it (M.S.); 7 Unit of Laboratory and Advanced Molecular Diagnostics, ‘Sant’Andrea’ University Hospital, 00189 Rome, Italy; 8 Department of Medical and Molecular Sciences, Faculty of Medicine and Psychology, Sapienza University, 00185 Rome, Italy; paolo.martelletti@uniroma1.it; 9 Headache Centre Unit, ‘Sant’Andrea’ University Hospital, 00189 Rome, Italy

**Keywords:** multiple chemical sensitivity, hyperosmia, asthenia, dyspnoea, respiratory symptoms, neurocognitive symptoms, anxiety, depression

## Abstract

Multiple Chemical Sensitivity (MCS) is a chronic and/or recurrent condition with somatic, cognitive, and affective symptoms following a contact with chemical agents whose concentrations do not correlate with toxicity in the general population. Its prevalence is not well defined; it mainly affects women between 40 and 50 years, without variations in ethnicity, education and economic status. We aimed to assess the core symptoms of this illness in a sample of Italian patients. Two physicians investigated different symptoms with a checklist compilation in 129 patients with MCS (117 women). We conducted a categorical Principal Component Analysis (CATPCA) with Varimax rotation on the checklist dataset. A typical triad was documented: hyperosmia, asthenia, and dyspnoea were the most common symptoms. Patients also frequently showed cough and headache. The CATPCA showed seven main factors: 1, neurocognitive symptoms; 2, physical (objective) symptoms; 3, gastrointestinal symptoms; 4, dermatological symptoms; 5, anxiety-depressive symptoms; 6, respiratory symptoms; 7, hyperosmia and asthenia. Patients showed higher mean prevalence of factors 7 (89.9%), 6 (71.7%), and 1 (62.13%). In conclusion, MCS patients frequently manifest hyperosmia, asthenia, and dyspnoea, which are often concomitant with other respiratory and neurocognitive symptoms. Considering the clinical association that is often made with anxiety, more studies are necessary on the psychosomatic aspects of this syndrome. Further analytical epidemiological studies are needed to support the formulation of aetiological hypotheses of MCS.

## 1. Introduction

Multiple chemical sensitivity (MCS) syndrome is a medically unexplained condition characterized by the appearance of symptoms in various organs and systems when the subject is exposed to odours of chemicals that are usually present in the environment at concentrations that are not related to hypersensitivity in the general population [1,2,3,4]. MCS patients related such reactions to exposure to such different chemicals as solvents, hydrocarbons, organophosphates, and heavy metals. MCS is currently included in the wider spectrum of idiopathic environmental intolerance (IEI), which also considers physical stimuli such as electromagnetic fields [5]. Common MCS symptoms are hyperosmia, weakness, lethargy, sore throat, dyspnoea, headache, and difficulties in concentration [1,5].

MCS aetiology and pathogenesis have not been clarified, as no variation in clinical trials has been reported. MCS prevalence is not well defined, probably since it is not a universally recognized diagnostic typology [6]; 40–50-year-old women are most affected [7,8], among whom there are no differences in ethnicity, education and economic status [9].

Currently, the World Health Organization has not recognized MCS as an organic, chemical-caused illness [3]. This condition has not been associated with objective pathophysiological processes [10], and many hypotheses have been formulated to explain its etiopathogenesis, including increase of histamine, involvement of immunological or inflammatory factors, psychological aspects, neurogenic inflammation, chronic hypoxia, and classical conditioning [11,12,13,14,15].

Different studies have suggested correlations of MCS symptoms with various possible triggers, including psychological and physical trauma, environmental exposure to chemical compounds, oxidative stress, and changes in the metabolism of xenobiotics related to genetic factors, including some variants of the CYP2D6, NAT2, PON1, MTHFR, and CCK2R genes [11,13,14,15,16,17,18,19,20]. Other studies have suggested a major role of the N-methyl-D-aspartate receptors [21,22], and changes in nitric oxide/peroxynitrite mechanisms [23,24].

The major hypothesis regarding the etiopathogenesis of MCS provides a multifactorial model, including genetic, environmental (exposure to chemical composites), and anamnestic (previous surgery) variables [20].

Cullen introduced the term “Multiple Chemical Sensitivity” in 1987, and drew up the following diagnostic criteria:

1. MCS is acquired in relation to some documentable environmental exposure that may initially have produced a demonstrable toxic effect.
2. Symptoms involve more than one organ system and recur and abate in response to predictable environmental stimuli.
3. Symptoms are elicited by exposures to chemicals even at very low concentrations.
4. No widely available test of organ system function can explain symptoms.

In 1996, the International Program of Chemical Safety (IPCS) of the World Health Organization proposed the Nethercott criteria [25]:

1. MCS is defined as a chronic condition (at least six months, causing significant impairment).
2. CNS symptoms are reproducible and associated with self-reported hypersensitivity.
3. Symptoms involve multiple systems (essential involvement of the CNS, and at least another system).
4. Symptoms occur with exposure to low levels of multiple (unrelated) chemicals and improve or remit when these triggers are avoided.

In 2005, an extension of the Nethercott criteria was proposed by Lacour & Zunder [26] and underlined the characteristic hypersensitivity to odours that follows the development of symptoms.

A new concept is the so-called “Central Sensitivity Syndromes”, which include the MCS, Chronic Fatigue Syndrome and Fibromyalgia, i.e., syndromes with considerable overlap of symptoms and mutual comorbidities [27,28]. These illnesses could have common pathophysiological mechanisms based on central sensitization [29,30].

For some authors, MCS is an unrecognised psychiatric disorder [31] or a psychosomatic disorder [32]. This is also based on the increased likelihood of MCS patients having a history of a psychiatric disorder and high rates of psychiatric comorbidities, including depression and anxiety, histrionic personality disorder, panic attacks, and trauma and childhood abuse [33,34,35].

On these bases, our main hypothesis was that MCS might show specific categories of symptoms, based on the organs/systems involved. The aim of this study was to identify the core MCS clusters of symptoms in a group of Italian patients attending a regional referral centre. The objectives consist in deepening knowledge of the main clinical aspects of the syndrome, dividing this into defined and identifiable dimensions, and opening the way to personalized treatments.

## 2. Materials and Methods

This was a single-centre, descriptive, observational, epidemiological study conducted at the Centre of Personalized Medicine at “Sant’Andrea” University Hospital, Rome, Italy.

Descriptive observational studies can reveal the frequency and distribution of health events in populations or groups of individuals. These are useful in bringing attention to characteristics of individuals suffering from a disease, or who have a greater risk of contracting a disease. They can also provide essential information for planning health care facilities. They represent a fundamental step towards proceeding with the formulation of etiological hypotheses that can be further verified through analytical epidemiological studies.

We analysed medical reports of patients who have had access to the centre for a medical consultation. We included data from patients who met the following criteria: MCS syndrome according to the Cullen criteria [1] with Lacour & Zunder revision [26], age >18 years. Exclusion criteria included pregnancy, recent brain injury, substance use, severe medical illness, and diagnosis of schizophrenia-spectrum disorders or acute psychiatric disorders.

Patients were assessed for MCS symptoms with a simple checklist compiled by two physicians (Table 1). The study was approved by the local ethical committee (code CE57732020); all subjects gave their informed consent before their inclusion in the study, which followed the generally accepted ethical research standards of the Declaration of Helsinki [36].

Statistical Analyses. We analysed the clinical characteristics of the samples with one-way analysis of variance (ANOVA1way) for the continuous variables, and the *Chi*-square (*χ^2^*) test for categorical variables. We analysed the homogeneity of variance with Levene tests. We conducted a categorical Principal Component Analysis (CATPCA) with Varimax rotation. Cut-off for statistical significance was set at *p* < 0.05. All *p* values were two-tailed. We used SPSS Statistics 25.0 software (IBM Corp., Armonk, NY, USA) for all analyses.

## 3. Results

### 3.1. Sociodemographic and Clinical Features of the Study Sample

Study participants were 129 patients (mean age 51.58 years, SD = 11.34), including 112 women and 17 men affected by MCS. We summarize the main sociodemographic and clinical features of the sample in Table 2. There was a significant prevalence of women, and the gender subsamples did not differ for age. The most frequent symptoms were hyperosmia, asthenia, dyspnoea, cephalalgia (headache), cough, attention deficit, tachypnoea, sense of confusion, nausea, light-headedness, and sense of suffocation/choking. The less frequent symptoms were cystitis, chest tightness, palpitations, vomiting, diarrhoea, meteorism (tympanites), motor incoordination, hyporexia, pressure peaks, trembling, gastro-oesophageal reflux, and recurrent fever. We report the confidence intervals of the prevalence of the analysed symptoms in Table 2.

### 3.2. Principal Component Analysis of Symptoms

The CATPCA showed seven components with Eigenvalue >1, the first of which explained 42.23% of the variance, the second 8.5%, the third 6.86%, the fourth 6.42%, the fifth 4.63%, the sixth 4.1%, and the seventh 2.74%. The seven factors accounted for 75.48% of the total variance.

The scree-test did not indicate the need to reduce the number of factors. The elements constituting each factor showed a high linear correlation, and Cronbach’s alpha was compatible with an excellent internal consistency of the checklist (0.99). Cronbach’s alphas for each Factor were 0.96 for Factor 1 (F1), 0.7 for Factor 2 (F2), 0.62 for Factor 3 (F3), 0.6 for Factor 4 (F4), 0.43 for Factor 5 (F5), 0.35 for Factor 6 (F6), and 0.01 for Factor 7 (F7).

From the analysis of the items included in each factor we proceeded to the following denominations: F1, neurocognitive symptoms; F2: physical (objective) symptoms; F3: gastrointestinal symptoms; F4: dermatological symptoms; F5: anxiety-depressive symptoms; F6: respiratory symptoms; F7: hyperosmia and asthenia. Analysing the expression of these factors, our sample showed a high mean prevalence of F7 (89.9%), F6 (71.7%), and F1 (62.13%) (Table 3).

One-way ANOVA did not show gender differences in age and factor scores, i.e., the sum of symptoms composing each factor (Table 4).

## 4. Discussion

One of the main problems in defining MCS based on its clinical presentation is the variation in individual responses to chemicals, which is mutable to the point that a characteristic pattern of symptoms has not been yet established [2]. The most common MCS symptoms reported in the literature are headache, dizziness, seizures, heart arrhythmia, gastrointestinal problems, breathing difficulties, fatigue, lethargy, sore throat, hyperosmia, dyspnoea, confusion, difficulty concentrating, and asthma attacks [5,37]. Patients usually refer these symptoms to different organs and systems and relate them to exposure or contact with one or more chemicals [38].

In line with existing evidence [7,8], we found that females showed higher prevalence than males; the most frequent symptom was hyperosmia, reported by 96.9% of the sample, followed by asthenia showed in 82.9% of patients. Other frequent symptoms were dyspnoea, cough, and cephalalgia. We found that hyperosmia and asthenia were significantly grouped together, constituting the most represented cluster of symptoms, followed by respiratory, and neurocognitive symptoms.

Hyperosmia and asthenia. Different studies showed that olfactory hypersensitivity is one of the most frequent clinical complaints referred by MCS patients [26,39,40], with different biological correlates. For example, a positron emission tomography study by Hillert and colleagues showed hypoactivation of odour-processing brain regions in MCS patients compared to healthy controls. MCS was also related to an odorant-related hyperactivation of the anterior cingulate cortex, cuneus, and pre-cuneus [41]. Moreover, limbic hyperactivity due to sensitization caused by neurogenic inflammation has been shown by neuroimaging studies, which have suggested that patients with MCS process odours differently from the healthy population showing dysfunction in different areas including the thalamus, amygdala and hippocampus, with hyperactivation of the left inferior temporal gyrus and a decrease in activity of the frontal cortex [39,40,41,42]. In addition to an increased smell hypersensitivity and poor quality of life, MCS could impair smell cognitive faculties, mainly the identification of odours [43]. This evidence is in line with the extension of the Lacour criteria that established the characteristic hypersensitivity to odours as a primary criterion for the diagnosis of MCS [26].

Asthenia/fatigue is the other main symptom of MCS in our sample. Its prevalence has been involved in theorizing a common aetiology of multiple chemical sensitivity, chronic fatigue syndrome, and posttraumatic stress disorder, to the point that changes in nitric oxide/peroxy-nitrite metabolism has been viewed as a common etiological correlate [24]. In this regard, an overlap of about 90% between MCS and chronic fatigue syndrome has been considered relevant [27].

Respiratory symptoms. Our results support the fact that MCS sufferers display frequent manifestation of respiratory symptoms. In this regard, patients often refer to respiratory and allergy specialists and their MCS symptoms can be often misdiagnosed as asthma or allergic conditions, which can lead to vain medical investigations [7]. MCS patients showed immunological alterations, including high levels of IFN-gamma, IL-10, IL-8, MCP-1, VEGF, and PDGF [44]. Despite this, studies have failed to reveal significant changes to provide diagnostic immunological tests or to monitor disease progression [45]. Furthermore, chemical smells typically alter the respiratory behaviour of patients [46]. The hypothesis of altered function of the respiratory mucosa in MCS [47] led to the discovery of inflammatory correlates of the upper airways [48,49]. Disruption of nasal respiratory epithelial surfaces has been involved in possible transport of some chemical substances to the brain via the cribriform plexus. Alternatively, pathology of nasal mucosa might lead to olfactory-limbic hyper-reactivity related to a neuronal kindling response [50,51,52]. Furthermore, chronic hypoxia has been involved in MCS physiopathology [15].

Gastro-enteric symptoms. We showed that gastrointestinal symptoms are statistically grouped in MCS, in line with existing research that reported irritable bowel, gastroesophageal reflux [53], and gastrointestinal symptoms [54] in MCS patients. These aspects point to the need for the study of eating behaviours and physical activities in this population, with the aim of improving these aspects with personalized treatment strategies.

Neurocognitive symptoms. Our study showed that MCS includes a major component of neurocognitive symptoms, in line with previous evidence regarding concentration difficulties, headache, and other cognitive symptoms. Other frequently reported symptoms are paraesthesia, light-headedness, and mental confusion [7,55].

Most patients relate these symptoms to the toxic effects of environmental “chemicals”, although direct evidence of this is lacking. Other hypotheses considered hyperventilation responses after stimuli exposure and suggested that the resulting hypocarbia might account for symptoms, comparable with panic disorder. This has been partially supported by response to treatment with sodium lactate compared to placebo [56].

Neuropsychological assessments of cognitive functioning may be an indirect method for determining the state of health of the central nervous system and could be important in MCS patients diagnosed with certainty [57].

Other somatic symptoms. Our study showed that MCS sufferers might present with some physical symptoms, including arthro-myalgia/fibromyalgia, cystitis, recurrent fever, motor incoordination, trembling, pressure peaks, and dizziness. This cluster of symptoms is the least represented in our sample (38.88% of patients), although arthro-myalgia, fibromyalgia, and cystitis, if considered separately, were displayed by more than 45% of patients.

Some patients experience significant discomfort from one or more of these symptoms, as reported in the literature regarding myalgia [58,59], hemodynamic changes [60], and dizziness. Other evidence showed that some MCS patients could manifest dizziness-related vestibular decay [55].

Furthermore, there is a recognized overlap among unexplained clinical conditions, including MCS, fibromyalgia, interstitial cystitis, and others [61]. Fever is not a specific symptom, although patients affected by hay fever often reported feeling ill with exposure to pesticides, drying paint, and car exhaust [62].

Dermatological symptoms. Atopic dermatitis significantly increased in MCS sufferers between 2012 and 2015 in Japan [63], and was significantly related to MCS [64,65]. Furthermore, MCS individuals showed increased non-allergic cutaneous reactions [66]. Exposure to volatile organic compounds enhanced skin wheal responses induced by histamine in patients with MCS, while it failed to do so in controls or patients with eczema/dermatitis syndrome. This can indicate that exposure to volatile organic compounds might be related to neurogenic inflammation and concomitant histamine-induced responses [52,67]. On the other hand, this symptom cluster has been viewed as a subtype of functional disorder [68].

Anxiety-depressive symptoms. Some authors have argued that MCS diagnosis might be a misdiagnosis of an unrecognized psychiatric disorder [31,69] or a “psychosomatic” illness [32]. Patients with MCS have an increased likelihood of having a history of psychiatric disorder and a very high rate of psychiatric comorbidity [34,35]. MCS patients significantly showed higher rates of depression and anxiety than the general population, with more frequent lifetime diagnoses of Major Depressive Disorder, generalized anxiety, and panic attacks [31,70]. Furthermore, our results confirmed a significant overlap between somatoform symptoms and MCS, which can be linked with this often-reported comorbidity [35]. This close connection between MCS and psychiatric diseases could be related to alterations of the cellular detoxification pathways and consequent issues in the oxidoreductive balance that would create a state of cerebral neuroinflammation, a condition that seems to play a fundamental role in the aetiology of psychiatric disorders. This may be linked to existing evidence of the involvement of specific polymorphisms of genes, such as CYP2D6, NAT2, and PON1, both in cellular detoxification and pathophysiology of psychiatric disorders and MCS, although further studies on these aspects are needed [71,72,73,74].

Limitations. The results of this study should be viewed with caution due to the following limitations. The study lacks a control sample (healthy population) and the statistical analyses are incorrect for any organic comorbidities, which may have precluded the observation of important outcomes. Since this is not an experimental study, it did not allow the verification of whether the association of symptoms with the diagnosis is causal or not. However, the study is observational and descriptive in nature, aiming to illustrate the main symptoms of patients who have received a diagnosis of MCS. In addition, reports of patients with severe chronic organic illnesses, acute pathologies, and acute psychiatric disorders were excluded.

## 5. Conclusions

MCS is a widespread disease or medical condition associated with exposure to common chemical pollutants, more frequently affecting adult women, and with unknown aetiology. It is mainly characterized by hyperosmia and asthenia, which are often associated with respiratory and neurocognitive symptoms. Gastro-enteric, dermatological and psychiatric symptoms are also often present, while other somatic symptoms are less frequent. MCS is a complex syndrome, involving many organs/systems, often displaying psychosomatic and psychiatric aspects. Further analytical epidemiological studies are needed to support the formulation of aetiological hypotheses of MCS.

## Figures and Tables

**Table 1:** Checklist of symptoms.

Symptom	YES	NO
Anxiety	☐	☐
Arthromyalgia	☐	☐
Asthenia	☐	☐
Attention deficit	☐	☐
Cephalalgia (headache)	☐	☐
Chest tightness	☐	☐
Cough	☐	☐
Cystitis	☐	☐
Decision making deficit	☐	☐
Depression	☐	☐
Diarrhoea	☐	☐
Dizziness	☐	☐
Dyspepsia	☐	☐
Dyspnoea	☐	☐
Erythema	☐	☐
Fibromyalgia symptoms	☐	☐
Gastric pyrosis (heartburn)	☐	☐
Gastro-oesophageal reflux	☐	☐
Hyperosmia	☐	☐
Hyporexia (Decreased appetite)	☐	☐
Light-headedness	☐	☐
Meteorism (tympanites)	☐	☐
Motor incoordination	☐	☐
Nausea	☐	☐
Palpitation	☐	☐
Paraesthesia	☐	☐
Pressure peaks	☐	☐
Pruritus (itch)	☐	☐
Rash	☐	☐
Recurrent fever	☐	☐
Sense of confusion	☐	☐
Sense of suffocation/choking	☐	☐
Sleep disturbance	☐	☐
Tachypnoea	☐	☐
Trembling	☐	☐
Vomiting	☐	☐
Working memory deficit	☐	☐

**Table 2:** Sociodemographic and clinical characteristics of the study sample.

	Sample Number	Ratio	*χ* ^2^	*p*
Gender (m/f)	129	17/112	69.961	<0.001
	Mean age (years)	SD	F	*p*
Males	49.59	10.67	0.603	0.439
Females	51.89	11.46		
Whole sample	51.58	11.34		
Symptom	95% Confidence Interval (%)	Symptom	95% Confidence Interval (%)	
Hyperosmia	0.94–1	Pruritus	0.42–0.59	
Asthenia	0.76–0.89	Dizziness	0.38–0.55	
Dyspnoea	0.75–0.88	Rash	0.37–0.54	
Cough	0.66–0.81	Fibromyalgia	0.36–0.54	
Cephalalgia (headache)	0.64–0.8	Erythema	0.36–0.54	
Tachypnoea	0.63–0.79	Working memory deficit	0.36–0.54	
Attention deficit	0.61–0.78	Diarrhoea	0.34–0.51	
Sense of confusion	0.58–0.75	Meteorism	0.34–0.51	
Nausea	0.56–0.73	Motor incoordination	0.33–0.5	
Light-headedness	0.55–0.72	Palpitations	0.33–0.5	
Sense of choking	0.5–0.67	Chest tightness	0.27–0.45	
Dyspepsia	0.5–0.67	Vomiting	0.27–0.45	
Sleep disturbance	0.49–0.66	Hyporexia	0.25–0.42	
Paraesthesia	0.47–0.65	Pressure peaks	0.21–0.38	
Anxiety	0.46–0.64	Trembling	0.21–0.38	
Decision making deficit	0.45–0.62	Gastro-oesophageal reflux	0.2–0.36	
Arthromyalgia	0.44–0.62	Cystitis	0.19–0.34	
Gastric pyrosis	0.42–0.6	Recurrent fever	0.16–0.32	
Depression	0.42–0.59			

**Table 3:** Categorical principal component analysis: rotated component matrix.

	F1	F2	F3	F4	F5	F6	F7	Item CI 95%	Mean Component CI
Light-headedness	0.880	0.205	0.119	0.043	0.095	0.030	0.078	0.55–0.72	
Sense of confusion	0.871	0.085	0.168	0.123	0.175	0.118	0.079	0.58–0.75	
Attention deficit	0.829	0.027	0.251	0.098	0.195	0.140	−0.035	0.61–0.78	
Decision making deficit	0.806	0.268	0.151	0.168	0.173	0.131	0.007	0.45–0.62	
Working memory deficit	0.675	0.564	0.088	0.029	0.096	0.067	−0.126	0.36–0.54	
Cephalalgia	0.648	0.059	0.088	0.112	0.294	0.215	0.316	0.64–0.8	0.53–0.7
Fibromyalgia	0.063	0.734	−0.026	0.052	−0.061	0.130	0.168	0.36–0.54	
Cystitis	0.064	0.718	0.255	0.373	−0.005	0.099	0.101	0.19–0.34	
Recurrent fever	0.108	0.711	0.336	0.314	0.152	0.029	0.030	0.16–0.32	
Trembling	0.226	0.690	0.200	0.097	0.384	0.017	0.070	0.21–0.38	
Motor incoordination	0.568	0.652	0.031	−0.025	0.090	0.050	0.103	0.33–0.5	
Pressure peaks	0.191	0.599	0.334	0.202	0.357	0.063	−0.054	0.21–0.38	
Dizziness	0.370	0.460	0.157	−0.022	0.439	0.142	0.289	0.38–0.55	
Arthromyalgia	0.254	0.446	0.163	0.114	0.226	0.245	0.436	0.44–0.62	0.29–0.45
Meteorism	0.224	0.261	0.788	0.145	0.038	0.087	0.020	0.34–0.51	
Dyspepsia	0.369	0.018	0.758	0.118	0.240	0.113	0.177	0.5–0.67	
Vomiting	0.031	0.361	0.720	0.115	0.233	0.177	−0.066	0.27–0.45	
Gastric pyrosis	0.354	0.032	0.711	0.331	0.259	0.179	0.012	0.42–0.6	
Diarrhoea	−0.082	0.260	0.681	0.076	0.052	−0.175	0.232	0.34–0.51	
Nausea	0.365	−0.041	0.627	0.103	0.384	0.104	0.257	0.56–0.73	
Gastro-oesophageal reflux	0.159	0.495	0.509	0.363	0.081	0.080	−0.120	0.2–0.36	0.38–0.55
Rash	0.069	0.240	0.190	0.870	0.198	0.193	0.009	0.37–0.54	
Erythema	0.091	0.234	0.203	0.866	0.182	0.186	0.080	0.36–0.54	
Pruritus	0.167	0.146	0.202	0.780	0.239	0.187	0.228	0.42–0.59	0.38–0.56
Anxiety	0.404	0.086	0.173	0.403	0.650	0.077	0.060	0.46–0.64	
Palpitations	0.214	0.347	0.311	0.208	0.631	0.086	−0.212	0.33–0.5	
Depression	0.336	0.073	0.223	0.477	0.625	0.103	0.041	0.42–0.59	
Chest tightness	0.214	0.479	0.319	0.225	0.545	0.092	−0.152	0.27–0.45	
Sleep disturbance	0.487	−0.017	0.397	0.260	0.528	0.140	0.177	0.49–0.66	
Hyporexia	0.236	0.377	0.401	0.252	0.492	0.227	0.077	0.25–0.42	
Paraesthesia	0.383	0.170	0.297	0.149	0.390	0.193	0.282	0.47–0.65	0.38–0.56
Dyspnoea	0.241	0.028	0.102	0.148	0.065	0.886	0.004	0.75–0.88	
Cough	0.147	0.095	0.044	0.302	0.071	0.765	0.014	0.66–0.81	
Tachypnoea	0.326	0.060	0.102	0.211	0.162	0.749	0.278	0.63–0.79	
Sense of choking	−0.209	0.495	0.059	−0.124	0.046	0.688	−0.090	0.5–0.67	0.64–0.79
Hyperosmia	−0.039	0.047	0.211	−0.012	−0.223	0.081	0.618	0.94–1	
Asthenia	0.243	0.132	−0.043	0.284	0.280	−0.067	0.670	0.76–0.89	0.85–0.95

Rotation Method: Varimax with Kaiser Normalization. F1: neurocognitive symptoms; F2: other somatic symptoms; F3: gastrointestinal symptoms; F4: dermatological symptoms; F5: anxiety-depressive symptoms; F6: respiratory symptoms; F7: hyperosmia and asthenia.

**Table 4:** Factor scores in gender subsamples (1-way ANOVA).

						95% CI			
	Gender	N	Mean	SD	SE	Lower Bound	Upper Bound	*F*	*p*
Age	Women	109	52.18	11.39	1.09	50.02	54.35	2.05	0.15
	Men	16	47.94	8.49	2.12	43.41	52.46		
	Total	125	51.64	11.12	0.99	49.67	53.61		
F1: neurocognitive symptoms	Women	109	5.29	3.52	0.34	4.62	5.96	0.00	0.98
	Men	16	5.31	3.28	0.82	3.56	7.06		
	Total	125	5.30	3.48	0.31	4.68	5.91		
F2: physical symptoms	Women	109	2.83	2.87	0.27	2.28	3.37	0.35	0.55
	Men	16	2.38	2.66	0.66	0.96	3.79		
	Total	125	2.77	2.83	0.25	2.27	3.27		
F3: gastrointestinal symptoms	Women	109	2.90	2.40	0.23	2.44	3.36	0.41	0.52
	Men	16	3.31	2.55	0.64	1.95	4.67		
	Total	125	2.95	2.42	0.22	2.52	3.38		
F4: dermatological symptoms	Women	109	1.48	1.48	0.14	1.20	1.76	1.92	0.17
	Men	16	0.94	1.29	0.32	0.25	1.62		
	Total	125	1.41	1.46	0.13	1.15	1.67		
F5: anxiety-depressive symptoms	Women	109	2.28	2.12	0.20	1.87	2.68	2.24	0.14
	Men	16	1.44	1.86	0.47	0.45	2.43		
	Total	125	2.17	2.10	0.19	1.80	2.54		
F6: respiratory symptoms	Women	109	2.89	1.46	0.14	2.61	3.17	0.70	0.41
	Men	16	2.56	1.50	0.38	1.76	3.36		
	Total	125	2.85	1.46	0.13	2.59	3.11		
F7: hyperosmia and asthenia	Women	109	1.78	0.48	0.05	1.69	1.87	0.59	0.44
	Men	16	1.88	0.34	0.09	1.69	2.06		
	Total	125	1.79	0.46	0.04	1.71	1.87		

Legend. CI: confidence interval; F1: neurocognitive symptoms; F2: other somatic symptoms; F3: gastrointestinal symptoms; F4: dermatological symptoms; F5: anxiety-depressive symptoms; F6: respiratory symptoms; F7: hyperosmia and asthenia; SD: standard deviation; SE: standard error.
